# Comparative assessment of insecticide resistance phenotypes in two major malaria vectors, *Anopheles funestus* and *Anopheles arabiensis* in south-eastern Tanzania

**DOI:** 10.1186/s12936-020-03483-3

**Published:** 2020-11-11

**Authors:** Polius G. Pinda, Claudia Eichenberger, Halfan S. Ngowo, Dickson S. Msaky, Said Abbasi, Japhet Kihonda, Hamis Bwanaly, Fredros O. Okumu

**Affiliations:** 1 Environmental Health and Ecological Sciences Department, Ifakara Health Institute, Morogoro, United Republic of Tanzania; 2 Nelson Mandela African Institution of Science and Technology, School of Life Sciences and Biotechnology, Arusha, United Republic of Tanzania; 3 School of Public Health, University of the Witwatersrand, Parktown, South Africa; 4 Swiss Tropical and Public Health Institute, Basel, Switzerland; 5 Institute of Biodiversity, Animal Health and Comparative Medicine, University of Glasgow, Glasgow, UK

**Keywords:** Insecticide resistance, *Anopheles funestus*, PBO, Ifakara health institute, Tanzania

## Abstract

**Background:**

Long-lasting insecticide-treated nets (LLINs) and indoor residual spraying (IRS) have greatly reduced malaria transmission in sub-Saharan Africa, but are threatened by insecticide resistance. In south-eastern Tanzania, pyrethroid-resistant *Anopheles funestus* are now implicated in > 80% of malaria infections, even in villages where the species occurs at lower densities than the other vector, *Anopheles arabiensis*. This study compared the insecticide resistance phenotypes between the two malaria vectors in an area where pyrethroid-LLINs are widely used*.*

**Methods:**

The study used the World Health Organization (WHO) assays with 1×, 5× and 10× insecticide doses to assess levels of resistance, followed by synergist bioassays to understand possible mechanisms of the observed resistance phenotypes. The tests involved adult mosquitoes collected from three villages across two districts in south-eastern Tanzania and included four insecticide classes.

**Findings:**

At baseline doses (1×), both species were resistant to the two candidate pyrethroids (permethrin and deltamethrin), but susceptible to the organophosphate (pirimiphos-methyl). *Anopheles funestus*, but not *An. arabiensis* was also resistant to the carbamate (bendiocarb). Both species were resistant to DDT in all villages except in one village where *An. arabiensis* was susceptible. *Anopheles funestus* showed strong resistance to pyrethroids, surviving the 5× and 10× doses, while *An. arabiensis* reverted to susceptibility at the 5× dose. Pre-exposure to the synergist, piperonyl butoxide (PBO), enhanced the potency of the pyrethroids against both species and resulted in full susceptibility of *An. arabiensis* (> 98% mortality). However, for *An. funestus* from two villages, permethrin-associated mortalities after pre-exposure to PBO only exceeded 90% but not 98%.

**Conclusions:**

In south-eastern Tanzania, where *An. funestus* dominates malaria transmission, the species also has much stronger resistance to pyrethroids than its counterpart, *An. arabiensis,* and can survive more classes of insecticides. The pyrethroid resistance in both species appears to be mostly metabolic and may be partially addressed using synergists, e.g. PBO. These findings may explain the continued persistence and dominance of *An. funestus* despite widespread use of pyrethroid-treated LLINs, and inform new intervention choices for such settings. In short and medium-term, these may include PBO-based LLINs or improved IRS with compounds to which the vectors are still susceptible.

## Background

Effective use of long-lasting insecticide-treated nets (LLINs) and indoor residual spraying (IRS) has tremendously reduced malaria transmission in sub-Saharan Africa [1, 2]. Despite this reduction, malaria transmission continues in several areas, driven by mosquitoes that are either physiologically [3–5] or behaviourally resistant [6–10] to current insecticide-based interventions. Resistance to commonly used pyrethroids has also necessitated a change of insecticide classes for IRS to either carbamates, organophosphates or more recently neonicotinoids approved by the World Health Organization (WHO) [11]. Similarly new insecticide-treated nets (ITNs) are being developed that contain either multiple insecticide classes [12] or pyrethroids and synergists [13, 14], and are expected to improve the control of resistant mosquitoes.

Mosquito resistance involves different mechanisms, through which they can withstand exposures to insecticides. These include metabolic resistance, target-site resistance, behavioural resistance, and cuticular resistance [15–17]. The discriminating concentration is used to evaluate the phenotypic resistance which when detected, the level of resistance can be subsequentlly determined by using intensity assays. The intensity assays uses the 5× and 10× discriminating concentration in a stepwise manner aiming at providing the information on the range of resistance present in a target vector [18]. Mosquitoes that express metabolic forms of resistance produce large quantities of enzymes or alternate the enzyme catalytic centre to efficiently detoxify the insecticide. The specific enzymes include monooxygenases (i.e. cytochrome P450s), which detoxify pyrethroids and carbamates, glutathione-S-transferases (GSTs), which detoxify organochlorides like DDT [17], and esterases, which detoxify pyrethroids and organophosphates [19, 20]. The degree to which the enzymatic proteins are expressed, and the level of resistance can be assessed using quantitative polymerase chain reaction (qPCR). Phenotypic assays use synergists, such as piperonyl butoxide (PBO), which enhance the potency of an insecticide by inhibiting the enzymes responsible for the insecticide metabolism [21]. On the other hand, some mosquitoes may have one or multiple target-site mutations due to modification of protein receptors usually targeted by insecticides (e.g. the voltage-gated sodium channels targeted by pyrethroids and organochlorides), thereby blocking or reducing the effectiveness of the insecticides [22–24]. Recently, scientists have also demonstrated that a sensory appendage protein (SAP2) enriched in the legs of malaria-carrying mosquitoes can also confer resistance to insecticides, thus allowing these mosquitoes to survive contact with ITNs [25].

Different *Anopheles* species have diverse levels of competencies in pathogen transmission, and also respond different to interventions based on their behaviour and physiology [26, 27]. With the rise of insecticide resistance in the vector populations, the choice of interventions will depend on characteristics of the local vectors. Comprehensive understanding of the distribution and underlying mechanisms of insecticide resistance is, therefore, important for planning and implementing vector control interventions.

In the villages of south-eastern Tanzania, where *Anopheles funestus* have been reported to be implicated in most of the ongoing malaria transmission [28–30], signs of resistance to most public health pesticides have been observed. *Anopheles funestus* also appears to survive longer than other co-existing vector species (parity rates are higher than *Anopheles arabiensis*) [28]. However, since the species is highly anthropophagic (prefers to blood-feed on humans over that of other vertebrates) [31, 32] and endophilic (prefers to bite indoors) [33], one would expect its populations and transmission activity to have been significantly reduced by ITNs now widely used in Tanzania for more than one decade [34–37]. Indeed, historical evidence from both East and Southern Africa suggests that effective insecticide-based indoor interventions can eliminate *An. funestus* on a local scale [38, 39]. *Anopheles gambiae *sensu stricto (*s.s.*), which is generally considered the most competent malaria vector, shares similar behaviours with *An. funestus*, i.e. high degree of anthropophily and endophilly [31, 32, 40]. However, *An. gambiae s.s.* unlike *An. funestus* has been highly impacted by ITNs in Kenya and Tanzania [41–43].

An important question, therefore, is why and how *An. funestus*, despite being highly anthropophagic and endophilic, survived the ITN onslaught, and why it continues to mediate most malaria transmission in rural south-eastern Tanzania despite co-occurrence with a different malaria vector species, *An. arabiensis*. One hypothesis has been that *An. funestus* expresses higher intensities of resistance to most of the commonly used insecticides in comparison to other malaria vectors and is, therefore, far less impacted by insecticidal interventions. A meta-analysis of various datasets has verified this phenomenon at global scale [44], but specific field tests comparing resistance intensities in the different vector species are limited. As shown by Kaindoa et al*.* in a study conducted in south-eastern Tanzania, *An. funestus* was resistant to pyrethroids, organochlorides, and carbamates [28]. Another study from the same study area demonstrated that resistance of *An. arabiensis* to diagnostic insecticide concentrations varied between nearby locations and seasons [45]. However, the intensity and mechanisms of these resistance phenotypes were not compared between species.

This study, therefore, compared the intensities and knockdown time of insecticide-resistance between the two main malaria vectors, *An. funestus* and *An. arabiensis*, in rural south-eastern Tanzania. Potential involvement of metabolic resistance and insecticide potency enhancement by Piperonyl Butoxide (PBO) synergists was also assessed.

## Methods

### Study site

**Fig. 1:**
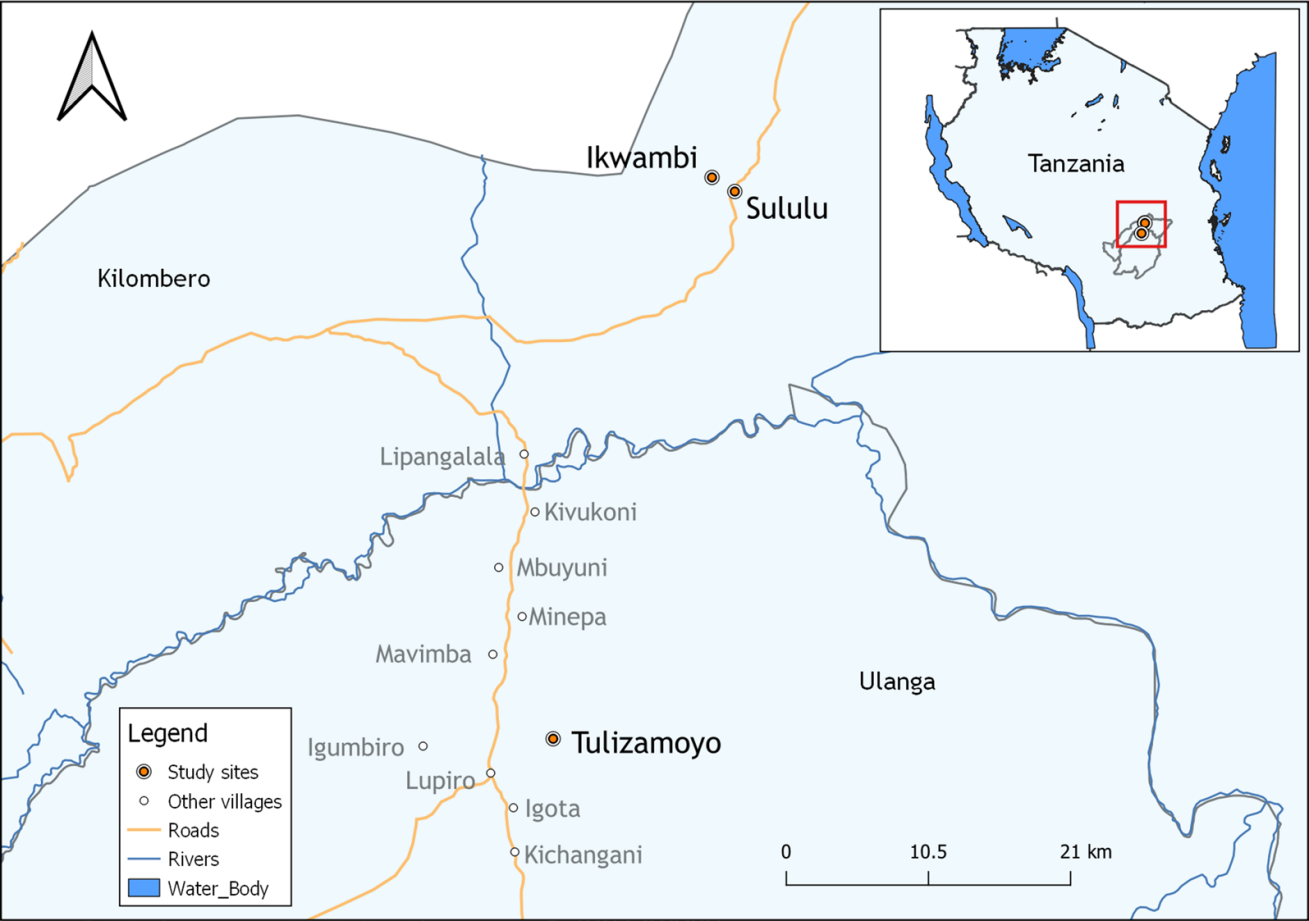
Locations of the study sites in Kilombero and Ulanga districts, where adult mosquito collections were performed

Mosquito collections were done in three different villages, namely; Ikwambi (7.98033°S, 36.81701°E) and Sululu (8.00324°S, 36.83118°E) in Kilombero district, and Tulizamoyo village (8.35747°S, 36.70664°E) in Ulanga district, south-eastern Tanzania (Fig. 1). The main malaria vectors in this area include *An. arabiensis* and *An. funestus,* with the latter driving more than 80% of the malaria transmission [28, 29]. This area has had high coverage of pyrethroid-treated nets for several years, but no IRS is implemented. The villages are all in low altitude areas, rising not more than 500 m above sea level. Mean daily temperatures are 20–33 °C, annual rainfall, 1200–1800 mm and relative humidity ranged between 24 and 97% [46, 47]. Most community members here are farmers, cultivating rice, maize and other crops in the Kilombero river valley.

### Mosquito collection

The WHO protocol for insecticide susceptibility tests [18] was used with slight modifications to conduct the basic bioassays and the resistance intensity assays. Since *An. funestus* mosquitoes were difficult to find as larvae across all the study villages, young nulliparous adult females of both *An. funestus* and *An. arabiensis* were used instead of larval collections. Mosquitoes were collected from September 2018 to November 2019 using CDC light traps [48]. Collections were done from 07.00 p.m. to 07.00 a.m. each night. To maximize probabilities of getting young unfed nulliparous females, houses near the edges of the villages and near potential habitats were selected for collections, based on previously-described heterogeneity of malaria transmission [49].

The traps were hung beside human occupied bed nets [50], but with extended catch bags to improve survival of the mosquitoes for subsequent assays. Each morning, after collections, the mosquitoes were transported to the Ifakara Health Institute’s mosquito biology laboratory, VectorSphere, in Ifakara and maintained at 27 ± 2 °C and 80 ± 10% relative humidity for 24 h to acclimatize as previously described [45]. During the acclimatization period, mosquitoes were supplied with 10% glucose solution. The mosquitoes were identified morphologically using the Gillies and Coetzee identification key [51], and non-target species were discarded. Tests were conducted using only non-blood-fed *An. funestus* and *An. arabiensis* females.

### Bioassays

This study involved three steps: (i) susceptibility assays to examine phonotypic resistance against standard insecticide doses, (ii) tests to assess intensity of resistance against chemicals to which resistance had been detected at baseline level, and (iii) tests using synergists to assess possible mechanisms of the observed reistance.

Baseline insecticide susceptibility bioassays were done according to WHO guidelines [18]. Candidate insecticides were selected from four classes as follows: organophosphate (0.25% pirimiphos-methyl), organochloride (4% DDT), carbamate (0.1% bendiocarb), pyrethroid type I (0.75%, 3.75 and 7.5% permethrin) and pyrethroid type II (0.05%, 0.25 and 0.5% deltamethrin). In each test, 120 mosquitoes were exposed to the insecticide-impregnated papers, and oil-impregnated papers as controls. Each test comprised six replicates (four treatments and two controls) with the total of 120 mosquitoes. Mosquitoes were exposed for 1 h and the knockdown time recorded at an interval of 10, 15, 20, 30, 40, 50, 60 min. They were then transferred to holding tubes, provided with 10% glucose solution, and their mortality recorded after 24 h.

Where resistance was observed in the baseline assays with standard diagnostic doses (i.e. 1×), additional tests were done to assess intensities of the resistance using 5× and 10× multiplicative doses of the insecticides. These included tests against 3.75% and 7.5% permethrin, and 0.25% and 0.5% deltamethrin. The procedures were similar to the baseline tests to assess the mortality.

Lastly, 4% Piperonyl Butoxide (PBO), a synergist, was used to assess the possible resistance mechanism by attempting to reverse the observed mortality outcomes [18]. Each test had four groups, each with 80 mosquitoes (in groups of 20), treated as follows: the first cohort was exposed to 4% PBO for one hour and immediately exposed to deltamethrin or permethrin for 60 min, a second group was exposed directly to the respective insecticides (i.e. deltamethrin, permethrin), a third group was exposed to the PBO only and the fourth group was exposed to control papers impregnated by silicone oil but no insecticide nor synergist. Given test kit limitations, the PBO tests were done only for pyrethroids.

### Molecular identification of sibling species of the tested mosquitoes

Up to 10% of the mosquitoes from each bioassay were packed separately and labelled with information about experimental date, village name, type of insecticide, insecticide dose used, species of mosquito, replicate number and sample ID. The packed mosquitoes were sent to the laboratory for molecular species identification of sibling species in the *An. funestus* and *An. gambiae* s.l. complexes, using DNA extracted from the mosquito legs. Polymerase chain reaction assays were conducted based on species-specific nucleotide sequences of the ribosomal DNA (rDNA) by relying on the intergenic spacer regions (IGS) for *An. gambiae *sensu lato (*s.l.*) members and the non-coding internal transcribed spacer 2 region (ITS2) for *An. funestus* [52, 53]. DNA bands were photographed under ultraviolet light using Kodak Gel Logic 100 imaging system [54].

### Data analysis

The data on insecticide susceptibility was interpreted based on the WHO-specified thresholds for resistance determination [18]. Susceptibility was confirmed when mortality was ≥ 98%, possible resistance was determined when mortality ranged from 90 to 97%, in which case the tests were repeated for confirmation, and resistance was confirmed when mortality was < 90%. When mortality greater than 10% was observed in controls, the test mortality was corrected using Abbott’s formula to avoid the biased estimations [55]. Tests were discarded and repeated, whenever control mortality exceeded 20% [18]. Final results were plotted in graphs using R software version 3.0 [56]. Log-probity analysis was used to calculate mean duration at which 50% (KDT_50_) and 95% (KDT_95_) of mosquitoes exposed to specific insecticides were knocked down.

## Results

### Phenotypic resistance at baseline insecticide concentrations

**Fig. 2:**
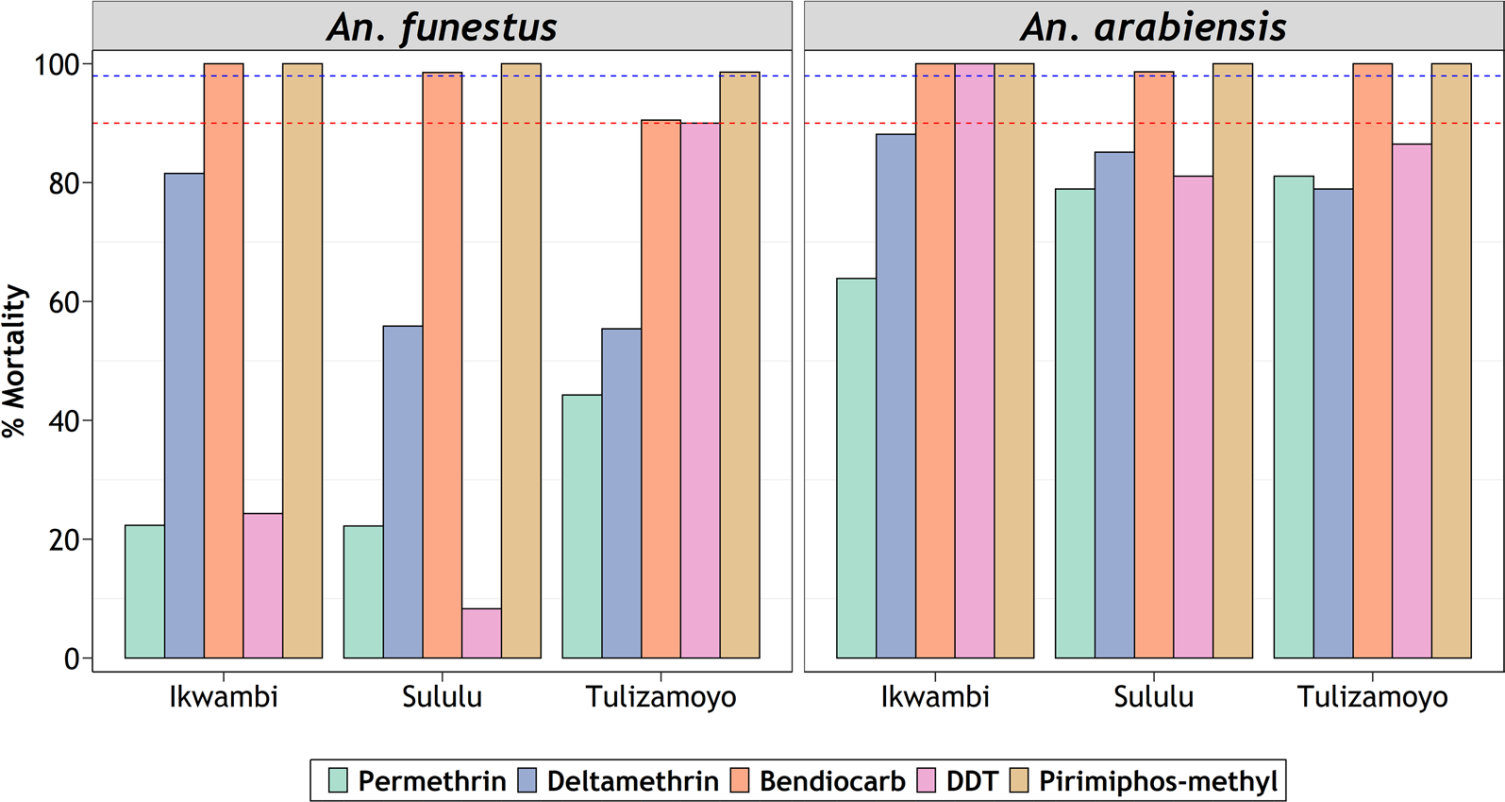
Percentage mortality of *Anopheles funestus* (right) and *Anopheles arabiensis* (left) exposed to baseline concentrations of candidate insecticides. Red-dotted and blue-dotted intercepts represent 90% and 98% mortalities indicative of resistance or susceptibility, respectively

Both species were resistant to the pyrethroids (permethrin and deltamethrin) and the organochloride (DDT), but susceptible to the organophosphate (pirimiphos-methyl) at standard baseline doses (1×). There was general susceptibility to the carbamate (bendiocarb) by both species across the study area, except in one of the villages, Tulizamoyo, where *An. funestus* were resistant to this insecticide. *Anopheles funestus* generally showed lower mortalities to the insecticides in the baseline tests compared to *An. arabiensis* (Fig. 2).

### Phenotypic resistance at 5 and 10 times baseline concentrations

**Fig. 3:**
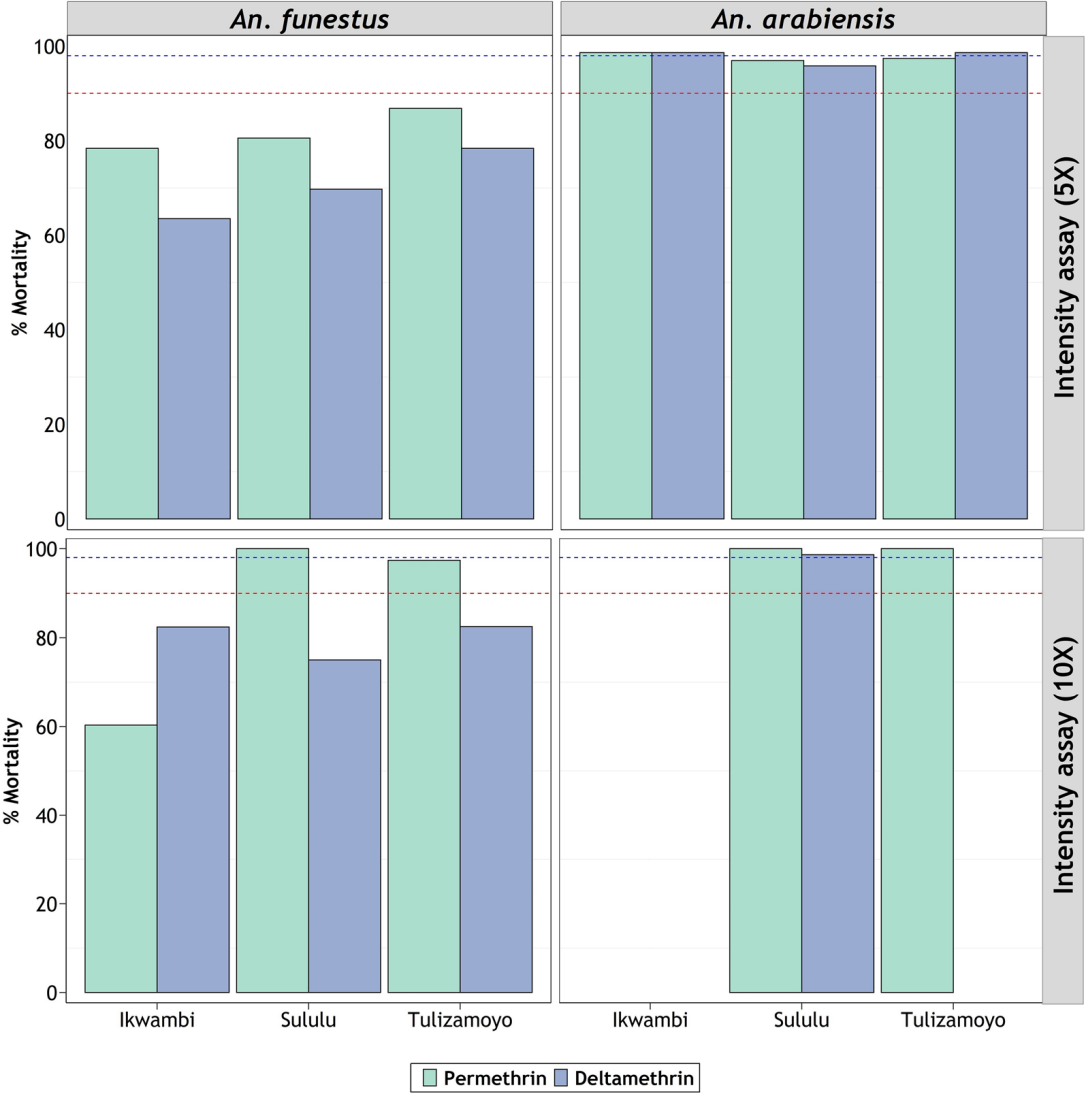
Resistance intensity of *Anopheles funestus* (right) and *Anopheles arabiensis* (left) under 5× and 10× baseline concentration. Red-dotted and blue-dotted intercepts represent 90% and 98% mortalities, respectively

*Anopheles funestus* populations from Ikwambi and Tulizamoyo are resistant to both 5× and 10× concentrations of permethrin, but the same species from Sululu were susceptible to 10× permethrin concentrations (Fig. 3). For *An. arabiensis* on the other hand, resistance intensity declined with increasing insecticide concentrations. Their resistance to pyrethroids was already overcome at 5× doses in Ikwambi and Tulizamoyo villages, while the ones from Sululu village, which survived 5× doses, were overcome at 10× doses. At 10× doses, *An. arabiensis* from all the villages were completely susceptible to the two pyrethroids (Fig. 3). Because of the observed susceptibilities at baseline doses (Fig. 1), no intensity assays were done against pirimiphos-methyl or bendiocarb on either of the species.

### Knockdown times (KDT)

Knockdown time for both mosquito species varied between insecticide and study villages. Consistently, the knockdown time slowed as the concentration increased for pyrethroid-exposed mosquitoes. Prolonged KDT_95_ (51–245 min) was observed to *An. funestus* exposed to pyrethroids compared to *An. arabiensis* (16–76 min), similar trend was observed on KDT_50_. Furthermore, the lowest knockdown time was observed when the mosquitoes were exposed to bendiocarb both at KDT_50_ and KDT_95_ for *An. funestus* and *An. arabiensis* (Table 1).

**Table 1:** The knockdown times of *An. funestus* and *An. arabiensis* mosquitoes at different insecticide concentrations

Insecticide	Village	Concentration	*An. arabiensis*		*An. funestus*	
		(Fold)	KDT_50_ ± SE (min)	KDT_95_ ± SE (min)	KDT_50_ ± SE (min)	KDT_95_ ± SE (min)
Deltamethrin	Ikwambi	1×	40.87 ± 12.55	76.68 ± 30.88	77.35 ± 50.19	128.13 ± 111.12
		5×	8.65 ± 6.70	23.34 ± 11.67	59.03 ± 22.43	99.19 ± 54.25
		10×	–	–	39.06 ± 12.85	77.21 ± 32.73
	Sululu	1×	38.81 ± 10.05	66.76 ± 21.76	86.39 ± 71.94	132.00 ± 146.02
		5×	11.61 ± 13.10	47.08 ± 23.83	68.15 ± 39.38	124.38 ± 97.13
		10×	8.63 ± 8.84	29.22 ± 15.50	45.94 ± 15.73	87.10 ± 41.42
	Tulizamoyo	1×	31.30 ± 8.82	56.48 ± 18.01	152.37 ± 388.44	245.22 ± 702.53
		5×	16.16 ± 5.75	29.99 ± 12.05	50.54 ± 19.60	96.49 ± 52.42
		10×	–	–	52.53 ± 16.60	88.72 ± 40.44
Permethrin	Ikwambi	1×	42.19 ± 10.82	71.25 ± 24.19	104.11 ± 124.69	177.35 ± 257.04
		5×	6.97 ± 5.17	16.15 ± 7.61	37.62 ± 11.07	70.12 ± 25.75
		10×	–	–	33.21 ± 11.87	71.14 ± 29.92
	Sululu	1×	25.35 ± 9.05	52.67 ± 19.02	82.76 ± 61.03	130.09 ± 127.92
		5×	4.05 ± 11.16	25.13 ± 15.64	33.26 ± 10.01	63.31 ± 22.11
		10×	7.83 ± 5.64	18.93 ± 8.92	20.21 ± 10.21	51.82 ± 21.66
	Tulizamoyo	1×	42.80 ± 11.56	73.95 ± 26.67	70.76 ± 41.28	124.31 ± 97.84
		5×	6.98 ± 8.14	24.26 ± 13.34	38.32 ± 13.87	80.67 ± 37.66
		10×	–	–	29.16 ± 8.61	53.82 ± 17.50
Pirimiphos-methyl	Ikwambi		49.06 ± 9.51	70.40 ± 20.85	32.31 ± 6.62	46.15 ± 11.89
	Sululu	1×	57.12 ± 10.92	75.62 ± 26.77	48.30 ± 7.54	64.09 ± 15.25
	Tulizamoyo		53.08 ± 14.22	83.01 ± 33.48	34.03 ± 4.70	40.42 ± 7.92
DDT	Ikwambi		44.82 ± 9.38	67.70 ± 19.65	97.77 ± 116.62	146.48 ± 222.68
	Sululu	1×	55.00 ± 11.41	76.66 ± 27.24	72.33 ± 37.37	119.73 ± 82.09
	Tulizamoyo		61.06 ± 23.07	99.04 ± 54.65	69.53 ± 35.24	113.72 ± 80.51
Bendiocarb	Ikwambi		14.86 ± 4.43	24.27 ± 9.45	26.32 ± 6.75	42.21 ± 13.04
	Sululu	1×	19.29 ± 4.37	27.23 ± 9.40	25.18 ± 4.92	33.00 ± 8.65
	Tulizamoyo		16.74 ± 3.36	22.85 ± 7.14	40.42 ± 7.61	57.80 ± 14.09

*SE* standard error, *KDT*_*50*_ time taken for 50% of the tested mosquitoes to be knocked-down, *KDT*_*95*_ time taken for 95% of the tested mosquitoes to be knocked-down

### Effects of pre-exposure to the synergist, PBO

**Fig. 4:**
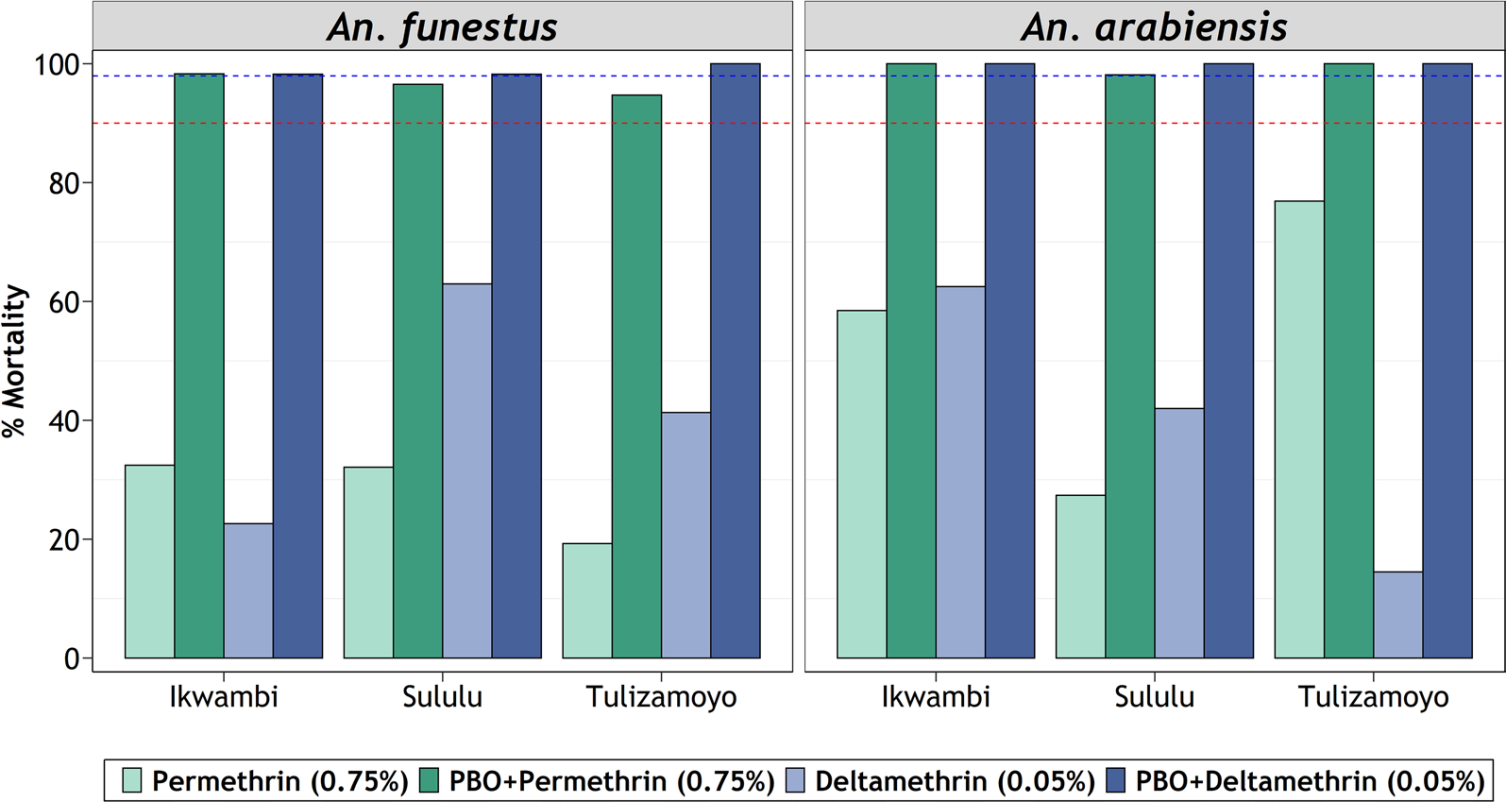
Proportion mortality of *Anopheles funestus* and *Anopheles arabiensis* to pyrethroids when pre-exposed to synergist. Red-dotted and blue-dotted intercepts represent 90% and 98% mortalities, respectively

Pre-exposure to the synergist, PBO, significantly reversed the pyrethroid resistance in both *An. arabiensis* and *An. funestus*. The PBO assays achieved mortalities > 98% in most cases, except for *An. funestus* populations from Sululu and Tulizamoyo villages, for which permethrin-associated mortalities were reversed past 95%, but not 98%. The synergist assays on *An. arabiensis* from all study areas demonstrated highest restoration of susceptibility (Fig. 4).

### Molecular identification of species

After the bioassays, a total of 305 *An. funestus* and 144 *An. arabiensis* were sent to the laboratory for sibling species identification. Of all the *An. funestus* assessed, successful PCR amplification was 76% (n = 233). Of those that amplified, 99% were *An. funestus s.s.* (n = 232), while one was amplified as *Anopheles leesoni*. For *An. gambiae s.l.* successful amplification was 92% (n = 132), all of which were identified as *An. arabiensis*. The rest did not amplify in the PCR assays (n = 12).

## Discussion

In this study, both *An. arabiensis* and *An. funestus* were resistant to pyrethroids and DDT. However, *An. funestus* exhibited far lower mortalities when subjected to pyrethroids at either the baseline concentration, five times concentration or the ten times concentration in the intensity bioassays. This suggests that while *An. funestus* is strongly resistant to the pyrethroids, the level of resistance in *An. arabiensis* was either low or moderate. This is the first study to directly compare resistance intensities of these two vectors in the area, and therefore provides important information on potential performance of current or future interventions against malaria. Given the differential contribution of the two vectors to overall transmission, their responsiveness to insecticidal interventions is an important factor for consideration in the elimination efforts.

Initial findings from standard WHO susceptibility assays by Kaindoa et al. [28] and Matowo et al. [45] on the two malaria vectors in the same study area observed that the baseline mortalities were higher in *An. arabiensis* than *An. funestus*. This was the initial indication that the intensity of resistance would be different between the two species, and necessitated additional tests according to standard WHO assays [18]. The duration at which either 50% or 95% of mosquitoes would be knocked-down varied between species, insecticides and study villages (Table 1).

Knock-down time was high when mosquitoes were exposed to standard concentrations of pyrethroids, but slowed as the concentrations increased. Since no *kdr* mutation tests were done, it is not possible to determine whether these observations were associated with the voltage-gated sodium channel protein mutation [57]. The slowest knockdown time was observed when the mosquito species were exposed to bendiocarb, these findings still support the potency of the insecticide (Table 1). The new findings clearly demonstrate that *An. funestus* populations, despite being the more dominant vector of malaria in the area, would be much more difficult to control using current pyrethroid-based interventions, in particular the LLINs.

As demonstrated by Matowo et al*.* for both *An. arabiensis* and *Culex* mosquitoes [45, 58], there were signs of fine-scale spatial variations in insecticide resistance. For example, *An. funestus* populations from Tulizamoyo were resistant to bendiocarb but populations of the same species from the other two villages were susceptible to the same chemical (Fig. 2). Similarly, the mortality percentages observed at 5× and 10× doses varied between the villages (Figs. 2, 3). This might be attributed to differences in the use of agricultural pesticides for crop protection in these villages [59]. Surprisingly, *An. arabiensis* from Ikwambi were 100% susceptible to DDT, against which both species from the other study villages were resistant (Fig. 2), which further suggests fine-scale spatial differences in resistance profiles.

The currently observed dominance of *An. funestus* is likely to be contributed by their well-documented resistance to commonly used insecticides [28, 60–64], their high survival probabilities in the wild [28, 30] and high levels of anthropophily [28, 31, 33]. Its dominance in areas where insecticidal interventions such as ITNs are widely implemented is particularly surprising given that scale-up of ITNs has coincided with significant declines in populations of other anthropophilic vectors such as *An. gambiae s.s.* [41–43]. Today, in rural south-eastern Tanzania, *An. funestus* co-exists with other *Anopheles* species, namely *An. arabiensis, An. leesoni, Anopheles coustani, Anopheles squamosus, Anopheles rivulorum* and *Anopheles pharoensis* [28]. However, it is known to carry most of the ongoing malaria transmission, sometime implicating to nearly nine in every ten new cases, even in areas where it occurs in lower densities than *An. arabiensis* [28, 29]. It was thus hypothesized that its dominance may at least be partly driven by stronger insecticide resistance levels to insecticides commonly used for public health, notably the pyrethroids used on bed nets.

As insecticide resistance increases across Africa, some populations have been observed to withstand up to 1000 times the standard concentrations [65], making it an urgent need to find new classes or combinations of insecticides [3–5, 17]. In areas where *An. funestus* is dominant, such as in south-eastern Tanzania, the decisions on which insecticides to be implemented in vector control measures should reflect intensity of resistance in this species, even if it is difficult to find its larvae. *Anopheles funestus* were resistant up to ten times the WHO-recommended concentration of pyrethroids, clearly indicating that this class of insecticides can no longer be useful in the area and must be urgently replaced by other classes such as organophosphates, against which resistance is not yet detected.

The synergist tests in this study showed complete or almost complete restoration of susceptibility in the malaria vector mosquitoes nearly from all study areas. This full restoration is a likely indicator of metabolic resistance [58, 66] and suggests that ITNs which have both PBO and pyrethroids, such as PermaNet 3.0 [14] and Olyset Plus [13], may be suitable for malaria prevention in these areas, and could potentially provide better protection than standard LLINs [67]. Synergist pre-exposure combined with deltamethrin had a greater restoration in *An. funestus* than when the synergist was combined with permethrin (Fig. 4), but in both cases there was still substantial restoration. This could be likely due to different resistance levels against the two pyrethroid classes as observed by Rakotoson et al*.* [68] when *An. arabiensis* were pre-exposed to PBO. Partial restoration of susceptibility observed in *An. funestus* mosquitoes might be a sign of multiple metabolic resistance forms or other resistance mechanisms including the target-site mutation [69]. This could also be a manifestation of the demonstrated high intensities of pyrethroid resistance (Fig. 3). These findings are in line with the previous studies on the resistance of malaria vectors to pyrethroids and organochlorides and incomplete susceptibility restoration after the synergist pre-exposure to pyrethroids [68]. Nonetheless, further exploration is needed to identify the specific metabolic enzymes responsible for the observed resistance under biochemical tests. Additionally, the level of these resistant enzymes needs to be assessed using quantitative PCR assays in both *An. arabiensis* and *An. funestus*.

Despite largely achieving the stated aims, the findings of this study should be considered only as indicative and not in any way conclusive. This is due to the various methodological limitations faced during the study. First, the overall collection of the specimen was distributed over several months, and may bave been influenced by seasonal variations in resistance, as previously demonstrated [45]. Besides, given the scarcity of *An. funestus* specimen in some of the villages, the tests used just 120 mosquitoes. Third, this study was the use of wild mosquitoes which may have varying ages, which is an important factor long-demonstrated to impact resistance [70–72].

While this way of testing gives a true representation of the natural mosquito population in communities and their ability to withstand insecticidal interventions, it makes it difficult to compare the tests in a conclusive manner. The WHO guidelines recommend the use of age-synchronized F_1_ generation, 3–5 days old [18]. In this study the challenge was minimized by: (a) collecting the adult female mosquitoes at the edges of the village near potential aquatic habitats, thus maximizing the chances of getting young nulliparous mosquitoes [49], (b) adding an acclimatization period of mosquitoes for 24 h between the actual mosquito collection and the resistance tests, and (c) using the CDC light trap for mosquito collection, thereby capitalizing collection of nulliparous host-seeking mosquitoes [73–75]. In addition, the tests did not combine collections from multiple days, but instead used synchronized days for each replicate, thus ensuring that the mosquito ages were approximately similar. It is recognized however that these improvements slightly improved the tests but are not adequate to enable conclusive determination or comparison of resistance levels.

Another limitation was the non-amplification of the samples where 8% (n = 12) of *An.arabiensis* and 24% (n = 62) of *An. funestus* complex were unidentifiable. It is possible that either there were polymorphisms in the ITS2 region of rDNA amplified in these assays, which might have been the main contributor of the observed non-amplification (Mapua et al., unpublished data), or there were a few other sibling species for which no primers were available in the assay. Future studies should involve a larger sample size, and possibly individual analysis of specimen to distinguish between species.

## Conclusion

Overall, this study has demonstrated that other than the differential importance of malaria vector species and the multiplicity of malaria transmission in different settings, the responsiveness of these vectors towards different insecticides may also vary. In rural south-eastern Tanzania, *An. funestus*, which now dominates malaria transmission, also indicate stronger resistance to pyrethroids commonly used on ITNs than its counterpart, *An. arabiensis*. Despite its rarity at aquatic stage, collection methods must endeavour to find this vector with synchronized age and study its resistance profile so that effective interventions can be mounted. Lastly, the study also emphasizes that decisions on which insecticidal interventions to apply should be informed by geographical and species-specific studies rather than generalized studies. In this cases, it appears that PBO-based LLINs and IRS with non pyrethroids, such as organophosphates may be appropriate for now, as the main vectors are still susceptible to these treatments.

## Data Availability

Data available upon request.

## Abbreviations

ITS2: Internal transcribed spacer 2 region
IGS: Intergenic spacer regions
IHI: Ifakara Health Institute
IRB: Institutional Review Board
LLINs: Long-lasting insecticidal nets
NIMR: National Institute for Medical Research
qPCR: Quantitative polymerase chain reaction
CDC: Centre for disease control and prevention
GSTs: Glutathione-*S*-transferase enzymes
